# MIR22HG regulates miR-486/PTEN axis in bladder cancer to promote cell proliferation

**DOI:** 10.1042/BSR20193991

**Published:** 2020-06-23

**Authors:** Qisheng Tang, Xue Jiang, Shanjin Ma, Lei Wang, Ruixiao Li, Jianjun Ma

**Affiliations:** 1 Department of Urology, Tangdu Hospital, Air Force Medical University, Xi'an City, Shaanxi Province 710038, China; 2 Operation Room, Department of Neurosurgery, Tangdu Hospital, Air Force Medical University, Shaanxi Province 710038, China

**Keywords:** bladder cancer, miR-486, MIR22HG, proliferation, PTEN

## Abstract

The tumor suppressive role of MIR22HG has been studied in several types of cancer. We analyzed the TCGA dataset and found the down-regulation of MIR22HG in bladder cancer (BC). Bioinformatics analysis predicted the interaction between MIR22HG and miR-486. The direct interaction between MIR22HG and miR-486 was also confirmed by dual luciferase assay. However, overexpression of these two factors did not significantly affect the expression of each other. Interestingly, overexpression of MIR22HG led to up-regulated phosphatase and tensin homolog (PTEN), which is a target of miR-486. In cell proliferation assay, overexpression of MIR22HG and PTEN led to decreased rates of BC cell proliferation. Moreover, overexpression of miR-486 played an opposite role and attenuated the effects of overexpression of MIR22HG and PTEN. Therefore, MIR22HG regulates miR-486/PTEN axis to promote cell proliferation in BC.

## Introduction

In clinical practice, bladder cancer (BC) is one of the most commonly diagnosed malignancies and a major cause of cancer mortalities [1]. According to the 2018 GLOBOCAN data, BC caused 199922 deaths, accounting for 2.1% of all cancer deaths [2]. In the same year, BC affected 549393 new cases, accounting for 3.0% of all new cancer cases [2]. Although the prognosis of BC has been significantly improved, the overall survival is still poor, owing to the low early diagnosis rate and the lack of effective therapies for patients at advanced stages [3,4]. Tobacco smoking and Schistosoma haematobium infections are major risk factors for BC [5], while these factors are not sufficient for the initiation of tumor, indicating the importance of the involvement of genetic factors [6,7].

Previous studies have identified a considerable number of genetic alterations involved in different aspects of BC development and progression [6,7]. Identification of the critical genetic players in BC may provide novel insights into the development of targeted therapies [8,9]. Phosphatase and tensin homolog (PTEN) is a well-studied tumor suppressive signaling that inactivates the main cancer cell survival pathway PI3K/Akt to regulate cell cycle and growth [10,11]. Some oncogenic miRNAs, such as miR-486, promote cancer cell proliferation by targeting PTEN [12]. Our preliminary bioinformatics analysis showed that miR-486 may bind with MIR22HG, a tumor suppressive long (>200 nt) non-coding RNA (lncRNA) in different types of cancer [13,14]. Based on our knowledge, the roles of MIR22HG in BC are unknown. By analyzing the TCGA dataset, we observed down-regulation of MIR22HG in BC. The present study was therefore carried out to explore the interactions between MIR22HG and miR-486 in BC.

## Materials and methods

### Patients and tissue collection

The present study passed the review of the Ethics Committee of Tangdu Hospital, Air Force Medical University. Paired BC and adjacent (2 cm around tumor) non-tumor tissue samples were collected from 58 BC patients (38 males and 20 females, 41–69 years old, mean age 54.5 ± 4.9 years old) through biopsy under the guidance of magnetic resonance imaging (MRI). All patients were diagnosed for the first time and no therapies were initiated before admission. No other clinical disorders were diagnosed in those patients. Based on AJCC staging system, the 58 patients included 12, 18, 15 and 13 cases at clinical stages I–IV, respectively. Histopathological exams were performed to confirm all tissue samples. All patients were informed of the experimental details and signed the informed consent.

### TCGA dataset analysis

The online program GEPIA (http://gepia.cancer-pku.cn/) was used to explore the expression data of MIR22HG in TCGA dataset. The function of ‘Single Gene Analysis’ was used with the inquiry of ‘MIR22HG’. Other parameters were default.

### BC cell line

Human BC cell line UMUC3 (ATCC, U.S.A.) was used. Cell culture conditions were 37°C, 95% humidity and 5% CO_2._ Cell culture medium was composed of 10% FBS and 90% Eagle's Minimum Essential Medium.

### Cell transfections

MiRNA negative control (NC) and miR-486 mimic were synthesized by Ribobio (Guangzhou, Chia). Expression vectors of MIR22HG and PTEN were constructed using pcDNA3.1 purchased from Sigma–Aldrich (U.S.A.) as backbone. Cells were harvested at 70–80% confluence and all transfections were mediated by lipofectamine 2000 (Life Technologies, U.S.A.). The dosages of miRNA (NC miRNA as NC group) and expression vector (empty vector as NC group) were 50 and 10 nM, respectively. Cells without transfections were cultivated until the end of experiment and were used as the Control (C) cells. Subsequent assays were performed using cells harvested at 48 h post-transfection.

### Dual luciferase reporter assays

Full length of MIR22HG cDNA was inserted into psiCHECK‐2 luciferase reporter vector (Promega, U.S.A.). Cells were transfected with MIR22HG vector combined with miR-486 mimic (miR-486 group) or miRNA NC (NC group). At 48 h after transfection, cell lysates were prepared and dual luciferase reporter assay kit (Promega) was used to measure luciferase activities following the manufacturer's instructions.

### RNA and RT-qPCR

TRIzol reagent (Invitrogen) was used to perform all RNA extractions. RNA samples were precipitated and washed with 85% ethanol to retain miRNAs. RQ1 RNase-Free DNase (Promega, U.S.A.) was used to digest RNA samples to remove genomic DNA. Digested RNA samples were reverse transcribed into cDNAs using M-MLV reverse transcription system (Promega) with poly (T) as the primer. All PCR reaction mixtures were prepared using ChamQ Universal SYBR qPCR Master Mix (Vazyme Biotech, China). The expression levels of MIR22HG and PTEN were measured with GAPDH as endogenous control. All-in-One™ miRNA qRT-PCR Detection Kit (QP015, Genecopoeia) was used to measure the expression levels of mature miR-486 with U6 as endogenous control. The fold changes of gene expression across samples were calculated using 2^−ΔΔCt^ method.

### Western blotting

Cell lysates were prepared using RIPA buffer (Beyotime), followed by centrifugation at 12000 ***g*** for 10 min to prepare protein samples. BCA assay (Beyotime) was used to measure protein expression levels. Denaturation of protein samples was performed in boiling water for 10 min, followed by SDS-PAGE gel to separate protein samples. Blocking was performed in TBST containing 5% BSA, followed by incubation at 4°C overnight with GAPDH (ab9845, Abcam) and PTEN (ab31392, Abcam) rabbit polyclonal primary antibodies. After that, membranes were further incubated with HRP Goat Anti-Rabbit (IgG) (ab6721, Abcam) secondary antibody at 25°C for 2 h. Signals were produced using ECL™ Western Blotting Analysis System (Sigma-Aldrich, U.S.A.). Image J v.1.48 was used to normalize signals.

### CCK-8 assay

CCK-8 assay was performed using Cell Counting Kit-8 kit (KeyGEN Biotech) to evaluate the effects of transfections on the proliferation of cells. Briefly, a 96-well plate was used to cultivate cells harvested at 48 h post-transfection with 3000 cells in 100 µl cell suspension per well. Three replicate wells were set for each transfection group. Cells were cultivated under aforementioned conditions and CCK-8 was added with 10 µl per well at 4 h before the end of cell culture. Optical density (OD) values were measured at 450 nm every 24 h until 96 h.

### Data analysis

Means ± SD was used to express the data from three independent replicates included in each experiment. Paired *t* test was used to explore the differences in the expression levels of MIR22HG between BC and non-tumor tissues. Paired *t* test was used to find differences between two groups. Differences among more than three groups were explored using ANOVA (one-way) and Tukey test. *P* < 0.05 was considered as statistically significant.

## Results

### MIR22HG was down-regulated in BC

**Figure 1:**
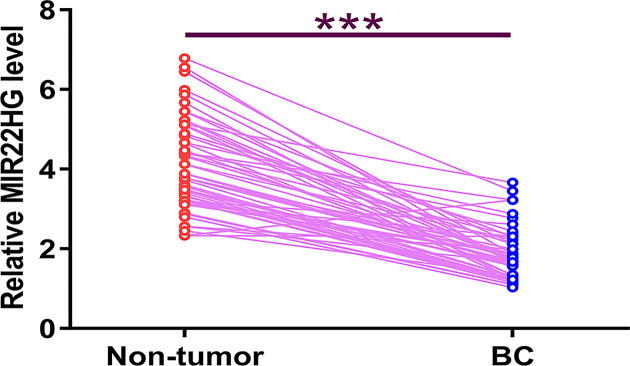
MIR22HG was down-regulated in BC Differential expression of MIR22HG in BC was explored by measuring its expression level in both BC and non-tumor tissues from the 58 patients included in the present study. PCR reactions were repeated three times and data were expressed as mean values. *, *P*<0.0001; ***, *P<0.05*.

TCGA is a useful dataset to analyze the differential expression of genes in different types of cancer. We first analyzed TCGA dataset and found that the expression levels of MIR22HG were obviously lower in BC tissues compared with that in non-tumor tissues (8.23 vs. 26.9). The differential expression of MIR22HG in BC was further explored by measuring its expression levels in both BC and non-tumor tissues from the 58 patients included in the present study. Compared with non-tumor tissues, the expression levels of MIR22HG were significantly lower in BC tissues (Figure 1, *P* < 0.0001).

### MIR22HG can directly interact with miR-486

**Figure 2:**
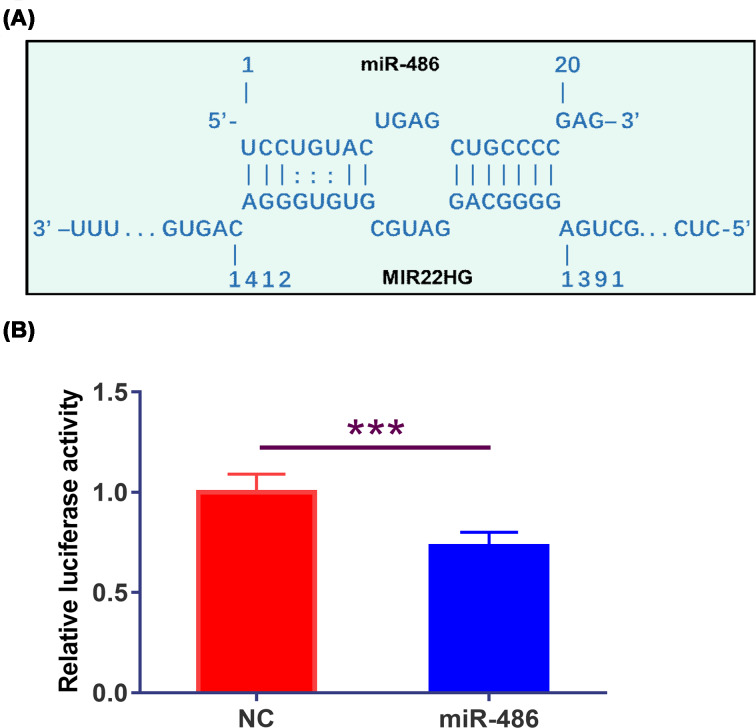
MIR22HG can directly interact with miR-486 IntaRNA (http://rna.informatik.uni-freiburg.de/IntaRNA/Input.jsp) was used to analyze the potential interaction between MIR22HG and miR-486. It was observed that miR-486 can bind MIR22HG (**A**). Dual luciferase assay was performed by transfecting MIR22HG vector + miR-486 (miR-486 group) or MIR22HG vector + miRNA NC (NC group) into UMUC3 cells, followed by the comparison of relative luciferase activity by performing unpaired *t* test (Figure 2**B**, *P* < 0.05). Mean values of three independent replicates were presented. *, *P*<0.05.

IntaRNA (http://rna.informatik.uni-freiburg.de/IntaRNA/Input.jsp) was used to analyze the potential interaction between MIR22HG and miR-486. It was observed that miR-486 can bind with MIR22HG (Figure 2A). Dual luciferase assay was performed by transfecting MIR22HG vector + miR-486 (miR-486 group) or MIR22HG vector + miRNA NC (NC group) into UMUC3 cells. Compared with NC group, the relative luciferase activity of miR-486 group was significantly lower (Figure 2B, *P* < 0.05).

### MIR22HG and miR-486 did not affect the expression of each other

**Figure 3:**
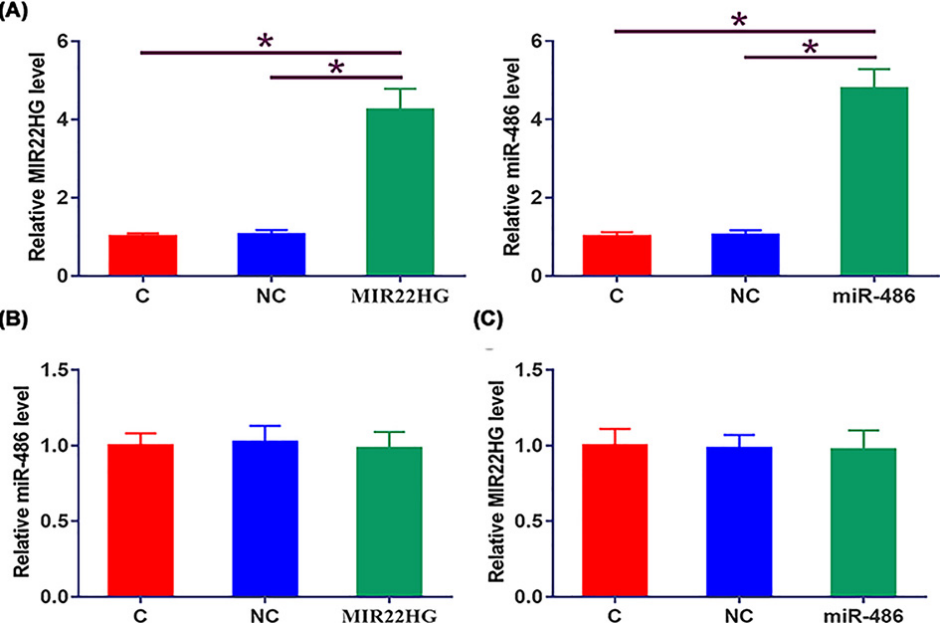
MIR22HG and miR-486 did not affect the expression of each other UMUC3 cells were transfected with MIR22HG vector or miR-486 mimic. Overexpression of MIR22HG and miR-486 was confirmed by qPCR at 48h post-transfection (**A**). The effects of MIR22HG overexpression on miR-486 (**B**) and the effects of miR-486 overexpression on MIR22HG (**C**) were explored by qPCR. Mean values of three independent replicates were presented. *, *P*<0.05.

UMUC3 cells were transfected with MIR22HG vector or miR-486 mimic. Overexpression of MIR22HG and miR-486 was confirmed by qPCR at 48 h post-transfection (Figure 3A, *P* < 0.05). Compared with NC and C groups, cells with overexpression of MIR22HG showed no significantly altered expression of miR-486 (Figure 3B). Moreover, overexpression of miR-486 also did not affect the expression of MIR22HG (Figure 3C).

### Overexpression of MIR22HG led to up-regulated miR-486 target PTEN

**Figure 4:**
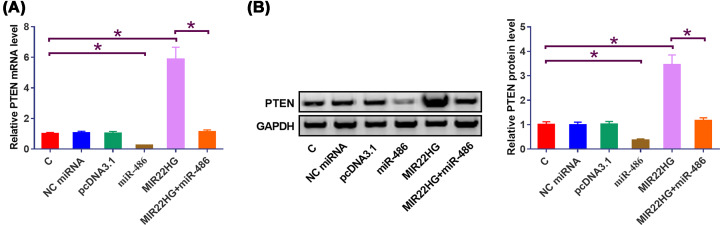
Overexpression of MIR22HG led to up-regulated miR-486 target PTEN The effects of overexpression of MIR22HG and miR-486 on the expression of PTEN at mRNA (**A**) and protein (**B**) levels were explored by qPCR and western blot, respectively. Mean values of three independent replicates were presented. *, *P*<0.05.

The effects of overexpression of MIR22HG and miR-486 on the expression of PTEN at mRNA (Figure 4A) and protein (Figure 4B) levels were explored by qPCR and western blot, respectively. Compared with cells transfected with empty pcDNA3.1 vector or miRNA NC (NC) and untransfected cells (C), cells with overexpression of MIR22HG showed significantly up-regulated PTEN (*P* < 0.05). Moreover, overexpression of miR-486 played an opposite role and attenuated the effects of overexpressing MIR22HG on the expression of PTEN (*P* < 0.05).

### MIR22HG suppressed UMUC3 cell proliferation

**Figure 5:**
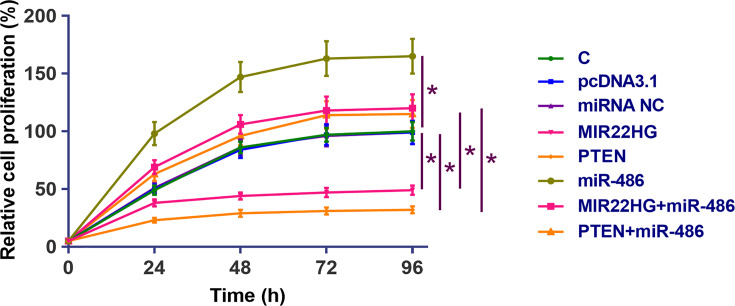
MIR22HG suppressed UMUC3 cell proliferation CCK-8 assay was performed to analyze the effects of MIR22HG, miR-486 and PTEN overexpression on the proliferation of UMUC3 cell proliferation. Mean values of three independent replicates were presented. *, *P*<0.05.

CCK-8 assay was performed to assess the effects of overexpression of MIR22HG, miR-486 and PTEN on the proliferation of UMUC3 cells. Compared with cells transfected with empty pcDNA3.1 vector or miRNA NC (NC) and untransfected cells (C), MIR22HG and PTEN overexpression resulted in decreased rates of BC cell proliferation. Moreover, overexpression of miR-486 played an opposite role and attenuated the effects of overexpressing MIR22HG and PTEN (Figure 5, *P* < 0.05).

## Discussion

No previous studies have explored the functions of MIR22HG in BC. We found that MIR22HG was down-regulated in BC. In addition, MIR22HG may suppress BC cell proliferation by regulating miR-486/PTEN axis.

Previous studies have characterized MIR22HG in different types of cancer. For instance, MIR22HG was down-regulated in hepatocellular carcinoma and its overexpression may affect miR-10a-5p/NCOR2 axis to suppress the invasion, migration and proliferation of cancer cells [13]. MIR22HG was also down-regulated in lung cancer and interacts with YBX1, MET and p21 to suppress cancer development [14]. By analyzing the TCGA dataset, we observed down-regulation of MIR22HG in most types of cancer including BC. We also confirmed the down-regulation of MIR22HG in BC tissues compared with non-tumor tissues. The only exception is glioblastoma (GBM). Analysis of TCGA dataset revealed the up-regulation of MIR22HG in GBM tissues compared with non-tumor tissues (11.06 vs. 7.51). Therefore, MIR22HG may play a different role in GBM. More studies are needed to verify this speculation.

MiR-486 is a well-established oncogenic miRNA in different types of cancer. Up-regulation of miR-486 not only affects cancer cell behaviors, such as proliferation [15], but also induces the development of chemoresistance during chemotherapy [16]. Based on our knowledge, the involvement of miR-486 in BC is still unknown. It has been reported that miR-486 can directly target PTEN to promote the proliferation of pancreatic cancer cells [12]. In this study we observed the down-regulation of PTEN and increased proliferation rate in BC cells with the overexpression of miR-486. In addition, miR-486 reduced the inhibitory effects of PTEN on cancer cell proliferation. Therefore, miR-486 may also target PTEN in BC to suppress cancer cell proliferation.

MIR22HG and miR-486 can interact with each other, while they did not affect the expression of each other. Interestingly, overexpression of MIR22HG led to the up-regulation of PTEN, a target of miR-486 in BC cells. Our data support the speculation that MIR22HG may be an endogenous sponge of miR-486. However, other mechanisms may exist.

In conclusion, MIR22HG was down-regulated in BC and may up-regulate PTEN by sponging miR-486 to suppress BC cell proliferation.

## Abbreviations

BC: bladder cancer
MRI: magnetic resonance imaging
NC: negative control
OD: optical density
PTEN: phosphatase and tensin homolog
